# Editorial: Advances in veterinary endocrine oncology

**DOI:** 10.3389/fvets.2024.1368962

**Published:** 2024-02-02

**Authors:** Floryne O. Buishand, Sara Galac

**Affiliations:** ^1^Clinical Science and Services, The Royal Veterinary College, Hawkshead Lane, Hatfield, United Kingdom; ^2^Department of Clinical Sciences, Faculty of Veterinary Medicine, Utrecht University, Utrecht, Netherlands

**Keywords:** endocrinology, endocrine oncology, oncology, pancreas, adrenal gland, pituitary gland, insulinoma

Endocrine oncology includes tumors of hormone-producing glands such as the pancreas, thyroid, parathyroid, pituitary and adrenal gland. Dogs and cats suffering from endocrine cancers benefit from a multidisciplinary approach by specialists in the fields of endocrinology, surgery, radiation therapy and nuclear medicine. The benefit of multidisciplinary teams is that they are able to provide a wide range of care from standard of care diagnostics, imaging studies and treatments to cutting-edge molecular diagnostics and state-of the-art surgical procedures.

Recent genomic studies have improved our understanding of underlying molecular mechanisms of certain endocrine cancers. For example, transcriptomic analysis by RNA-sequencing characterized malignant progression of canine insulinoma from normal tissue to metastatic disease (1). RNA-sequencing has also been used to identify potential novel therapeutic targets in canine cortisol-secreting adrenocortical tumors (2). Additionally, whole genome sequencing provided novel insight into the genetic causes of canine familial thyroid follicular cell carcinoma, which enabled the development of a genetic test to screen susceptible dogs (3).

Despite recent advances, the underlying mechanisms of tumorigenesis of endocrine cancers in dogs and cats are still largely unknown. A better understanding of the molecular background of these tumors could help to identify novel drug targets improving clinical outcome. The aim of this Research Topic was to provide readers with an update on the most recent pre-clinical and clinical advances in veterinary endocrine oncology.

The first article included in this Research Topic by Erger et al. reports on the clinicopathologic features and management of cats with androgen-secreting adrenal tumors. The authors argue that practitioners should be aware that androgenization in neutered cats resulting from androgen-secreting adrenal tumors could be the cause of unexplained urine marking or aggression that are poorly responsive to treatment. Furthermore, immunohistochemical assessment of adrenal enzymes and transcription factors that are deemed critical in adrenal androgen production is novel and superb and supports the diagnosis.

The second article by Stee et al. gives a detailed description of the surgical technique of transsphenoidal hypophysectomy in brachycephalic dogs. The authors emphasize the precautionary measures that need to be taken to safely perform this procedure in dogs with a brachycephalic skull conformation. Pre-operative *in silico* planning using computed tomography reconstructions and resection of part of the hard palate facilitate access to the burr hole site on the sphenoid bone.

The article by Bokhorst et al. compares the perioperative complications, success- and recurrence rates, long-term survival and prognostic factors in a cohort of 70 dogs with adrenal tumors treated by either laparoscopic or open adrenalectomy. The authors did not find significant differences between the laparoscopic and open adrenalectomy groups regarding peri-operative complication rate, recurrence, disease-free period, and survival time. Mean hospital stay was significantly shorter in the laparoscopic adrenalectomy group compared to the open adrenalectomy group. Therefore, the authors argue that the less invasive laparoscopic adrenalectomy is considered the preferred technique to treat dogs with adrenal tumors.

van den Berg et al. used RNA-sequencing for a transcriptomic analysis comparing canine pheochromocytomas and paragangliomas with normal canine adrenal medullas. They found overexpressed genes in canine pheochromocytomas and paragangliomas related to cell cycle, tumor development, progression and metastasis, hypoxia and angiogenesis, and the Wnt signaling pathway. Among the upregulated genes Ret Proto-Oncogene (*RET*), Dopamine Receptor D2 (*DRD2*) and Secreted Frizzled Related Protein 2 (*SFRP2*) could provide targets for novel therapeutics. Finally, their analysis also identified two transcriptionally distinct groups of canine pheochromocytomas and paragangliomas that had significantly different survival times.

The final article included in this Research Topic by Keulen and van Nimwegen describes a novel laparoscopic lateral flank approach for the minimal invasive partial pancreatectomy to treat canine insulinomas. This case series included four dogs from both small and large breeds with TNM stage 1 insulinomas in either left or right pancreatic limb. Laparoscopic procedures were performed without any major complications and survival times ranged from 599 to 1232 days.

Altogether, these manuscripts present new insights from current investigations focusing on endocrine cancers in companion animals with the specific aim to improve our current diagnostic and therapeutic approaches.

## Author contributions

FB: Writing – original draft, Writing – review & editing. SG: Writing – review & editing.
